# Identification of Potential Gene and MicroRNA Biomarkers of Acute Kidney Injury

**DOI:** 10.1155/2021/8834578

**Published:** 2021-01-08

**Authors:** Si-Yang Wang, Jie Gao, Yu-huan Song, Guang-Yan Cai, Xiang-Mei Chen

**Affiliations:** ^1^Department of Nephrology, The First Medical Centre, Chinese PLA General Hospital, Chinese PLA Institute of Nephrology, State Key Laboratory of Kidney Diseases, National Clinical Research Center for Kidney Diseases, Beijing 100853, China; ^2^953th Hospital, Shigatse Branch, Xinqiao Hospital, Army Medical University (Third Military Medical University), Shigatse 857000, China; ^3^Department of Nephrology, Shandong Provincial Hospital Affiliated to Shandong University, Jingwu Road 324, Jinan 250000, China; ^4^Department of Nephrology, Aerospace Center Hospital, 15 Yuquan Road Beijing 100049, China

## Abstract

Acute kidney injury (AKI) is a disease that seriously endangers human health. At present, AKI lacks effective treatment methods, so it is particularly important to find effective treatment measures and targets. Bioinformatics analysis has become an important method to identify significant processes of disease occurrence and development. In this study, we analyzed the public expression profile with bioinformatics analysis to identify differentially expressed genes (DEGs) in two types of common AKI models (ischemia-reperfusion injury and cisplatin). DEGs were predicted in four commonly used microRNA databases, and it was found that miR-466 and miR-709 may play important roles in AKI. Then, we found key nodes through protein-protein interaction (PPI) network analysis and subnetwork analysis. Finally, by detecting the expression levels in the renal tissues of the two established AKI models, we found that Myc, Mcm5, E2f1, Oip5, Mdm2, E2f8, miR-466, and miR-709 may be important genes and miRNAs in the process of AKI damage repair. The findings of our study reveal some candidate genes, miRNAs, and pathways potentially involved in the molecular mechanisms of AKI. These data improve the current understanding of AKI and provide new insight for AKI research and treatment.

## 1. Introduction

Acute kidney injury (AKI) is a group of common clinical syndromes of acute (a few hours to weeks) deterioration or even loss of kidney function due to various causes. The clinical manifestations range from mild kidney injury to renal failure. AKI has the characteristics of high morbidity and mortality, and there is no effective clinical treatment. Supportive care or dialysis treatment is usually performed, so medical expenses are high. In the absence of timely and effective treatment, AKI will eventually lead to death or progression to end-stage renal disease (ESRD). These issues have made AKI a major public health problem worldwide [1]. Therefore, it is particularly important to identify the pathological process of AKI to find new effective treatments for AKI.

The pathogenesis of AKI is multifactorial and complicated. Through the observation of two major AKI animal models (ischemia-reperfusion injury (IRI) and drug-induced damage), a variety of mechanisms are involved in the pathological process of AKI, such as mitochondrial dysfunction, autophagy, inflammation, and oxidative stress [2]. MicroRNA (miRNA) is a class of noncoding, single-stranded, small-molecule RNAs that can inhibit target mRNA translation or induce target mRNA degradation, thereby exerting posttranscriptional gene regulatory functions [3]. Studies in recent years have shown that through posttranscriptional gene regulation, miRNAs play important roles in kidney development and physiological conditions as well as in pathological conditions. By sequencing the AKI animal model, the expression of mRNA after AKI has been reported in several studies [4, 5]. Some studies have been conducted to observe the miRNA expression in the kidneys of an AKI model and to correlate the miRNA expression with the mRNA expression [6, 7]. However, the changes in miRNA and mRNA and their interactions in AKI pathology are still not fully understood. Moreover, the similarities and differences in miRNA and mRNA in AKI due to different underlying causes have not been verified. The Gene Expression Omnibus (GEO) database provides the opportunity for bioinformatics mining of the gene expression profiles. After conducting an interactive analysis on the GEO database datasets, we extracted a set of differentially expressed genes (DEGs) that are potentially involved in the progression of different AKI models (IRI and cisplatin (CIS)). By Gene Ontology (GO) and Kyoto Encyclopedia of Genes and Genomes (KEGG) pathway enrichment analyses, along with the construction of a protein-protein interaction (PPI) network and a miRNA-gene network, we identified the key genes, miRNAs, and the underlying signaling pathways playing a significant role in AKI development. Finally, we tested the DEGs and DE miRNAs in IRI and CIS-induced AKI models. By using these methods, our study is aimed at providing novel insight into the pathogenesis of AKI and identify novel therapeutic biomarkers for AKI caused by ischemia and drug toxicity.

## 2. Methods

### 2.1. Datasets

Two mRNA expression datasets (GSE106993 and GSE98622) were downloaded from the GEO database (https://www.ncbi.nlm.nih.gov/geo/). The GSE106993 dataset included eight samples (CIS group), including four CIS-induced AKI samples and four control [5]. The platform used was GPL21103. The GSE98622 dataset consisted of twelve samples (IRI group), including three kidney ischemia-reperfusion injury samples and nine control [4]. The time point of ischemia-reperfusion injury samples used in our analysis is 48 hours. The platform used was GPL13112. We standardized the data in each dataset and subjected the data to quality control analysis.

### 2.2. Identification of DEGs

Principal component analysis (PCA) executed in R software with the stats packages was used to obtain an intuitive distribution of the sample between the experimental and control groups. After the correlation analysis of samples and PCA, the calculation and screening of DEGs between the two groups were performed. DEGs were analyzed using the limma package, and adjusted *P* values < 0.01 and ∣fold change | >4 were used as cutoff criteria. A volcano map and clustering heat map were executed in R software with the ggplot packages, respectively. The fold change and *P* value between the groups for each gene in the two datasets were calculated.

### 2.3. Analysis of miRNA-mRNA Interactions

By analyzing the DEGs, the DEGs unique to the CIS model, the DEGs unique to the IRI model, and the overlapping DEGs shared by the CIS and IRI models can be separately obtained. DEGs of the three datasets were used to predict the miRNAs in the miRNA databases (miTarBase, miRanda, miRDB, and TargetScan) and construct a miRNA-mRNA interaction network. The miRNA-mRNA interaction pairs were visualized using Cytoscape.

### 2.4. GO and KEGG Pathway Enrichment Analyses

R software with the clusterProfiler packages was used to perform functional and pathway enrichment analyses of the DEGs with adjusted *P* values < 0.05.

### 2.5. PPI Network Construction and Module Analysis

This experiment used the STRING database (version 11.0) to detect PPIs of the proteins in three miRNA-mRNA regulatory networks. Required confidence (combined score) > 0.7 was the threshold for PPI in this study. Cytoscape was used to visualize the PPI network. The Molecular Complex Detection (MCODE) module with default parameters (degree cutoff = 2, node score cutoff = 2, *K*‐core = 2, and max depth = 100) and the CytoHubba module were used to explore the hub genes of the PPI network.

### 2.6. Animals

All animal procedures carried out in this study were approved by the Animal Care and Use Committee of Chinese PLA General Hospital and conducted in accordance with the Guide for the Care and Use of Laboratory Animals published by the US National Institutes of Health (NIH publication no. 85-23, revised 2011). Three-month-old C57BL/6 mice were purchased and housed at the Experimental Animal Center of the Chinese PLA General Hospital. For CIS-induced AKI, mice were intraperitoneally injected with a single dose of CIS at 20 mg/kg. The sham group was intraperitoneally injected with saline. After 72 hours, the mice were anesthetized with an intraperitoneal injection of sodium pentobarbital. Kidney ischemia was induced in mice by clamping the renal pedicle for 45 min to induce ischemia, and the kidney was subjected to reperfusion for 48 h. A midline abdominal incision was performed to expose both renal pedicles. Mice that underwent this procedure were used as sham surgery controls. The mice were anesthetized with an intraperitoneal injection of sodium pentobarbital, and all efforts were made to minimize suffering. Blood samples were taken, and their kidneys were removed.

### 2.7. RNA Isolation and Real-Time PCR

Total RNA was isolated from renal tissues using TRIzol (Invitrogen, Carlsbad, CA) according to the manufacturer's instructions. A UV spectrophotometer was used to measure the concentrations of total RNA. Reverse transcription was performed using a ProtoScript II First Strand cDNA Synthesis Kit (NEBNext). The miScript Reverse Transcription Kit (Qiagen) was used for cDNA preparation of miRNA. The reaction mixture comprised 50 ng of complementary deoxyribonucleic acid, 0.2 *μ*M primers, and 10 *μ*L of 2 × SYBR green buffer (Applied Biosystems, Foster, CA) in a final volume of 20 *μ*L. The miScript SYBR Green PCR Kit (Qiagen) was used for miRNA PCR. Relative quantification was performed by RQ manager software using the *ΔΔ*Ct method. The PCR analyses were conducted using an Applied Biosystems ABI Prism 7500 Sequence Detection System.

The relative levels of mouse E2f8 (sense: 5′-CGTCCCTCATCAAGTTGGTAAAG-3′, antisense: 5′-CCTGGGTTCACTTGACTGCTCTT-3′); mouse Oip5 (sense: 5′-GCCTTCTCCAAAGTCACAAACA-3′, antisense: 5′-AACCAACAGGAGTCCCACAGG-3′); mouse Msr1 (sense: 5′-CAGACTGAAGGACTGGGAACACT-3′, antisense: 5′-GTCCAGTAAGCCCTCTGTCTCC-3′); mouse Myc (sense: 5′-CCTAGTGCTGCATGAGGAGACA-3′, antisense: 5′-CTGTGCGGAGGTTTGCTGTG-3′); mouse Mdm2 (sense: 5′-GATGGCGTAAGTGAGCATTCTG-3′, antisense: 5′-AGACTGTGACCCGATAGACCTC-3′); mouse Mcm5 (sense: 5′-ACTCAAGCGGCATTACAACCT-3′, antisense: 5′-CGGCACTGAATGGAGATACGA-3′); and mouse E2f1 (sense: 5′-CGCACAGTTGCTTGTTGGAG-3′, antisense: 5′-TTGGTGGTCAGATTTAGTGAGGTT-3′) were normalized based on the level of 18 s. The relative levels of miR-466 k (5′-TGTGTGTGTACATGTACATGTGA-3′), miR-466a (5′-TATACATACACGCACACATAAGA-3′), miR-466f (5′-CATACACACACACATACACAC-3′), and miR-709 (5′-GGAGGCAGAGGCAGGAGGA-3′) were normalized based on the level of RNU-6B.

### 2.8. Statistical Analysis

All data were analyzed using SPSS 17.0 (SPSS, Chicago, IL). Comparisons among groups were analyzed by analysis of variance (ANOVA). Values of *P* < 0.05 indicated statistical significance.

## 3. Results

### 3.1. DEGs in Different AKI Models

**Figure 1:**
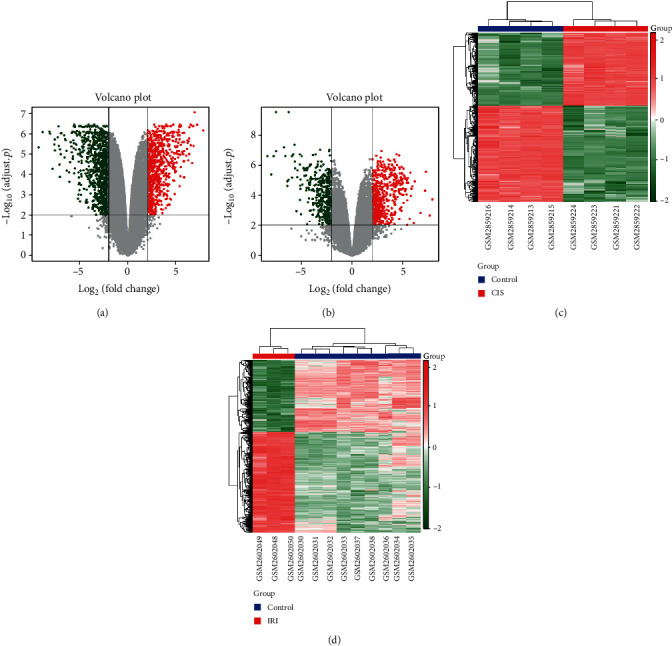
Differential gene expression patterns between AKI renal tissue and control tissue. (a) Volcano map of DEG expression levels between CIS-induced AKI and control samples. (b) Volcano map of DEG expression levels between IRI-induced AKI and control samples. The red nodes represent upregulated DEGs with *P* values < 0.01 and ∣log FC | >2; the green nodes represent downregulated DEGs with *P* values < 0.01 and ∣log FC | >2. (c, d) Heat map of the expression of DEGs.

The GSE106993 and GSE98622 datasets were downloaded from GEO, and the data were quality controlled and standardized with limma and stats package in R software. The DEGs in the CIS group and the control group were analyzed, and a total of 1756 DEGs were obtained, of which 998 had a low expression and 758 had a high expression. The DEGs between the IRI-48 h group and the control group were analyzed. A total of 969 DEGs were obtained, of which 402 had a low expression and 567 had a high expression. The screening criteria for DEGs were ∣fold change | >4, and the corrected *P* value was <0.01. We visualized DEGs using volcano maps (Figures 1(a) and 1(b)). Hierarchical clustering was used to cluster samples and genes according to the expression values of differential genes in different samples, and the expression of different genes in different samples was shown through a heat map (Figures 1(c) and 1(d)).

### 3.2. miRNA-mRNA Interaction Analysis of the Unique and Shared DEGs of the CIS and IRI Groups

**Figure 2:**
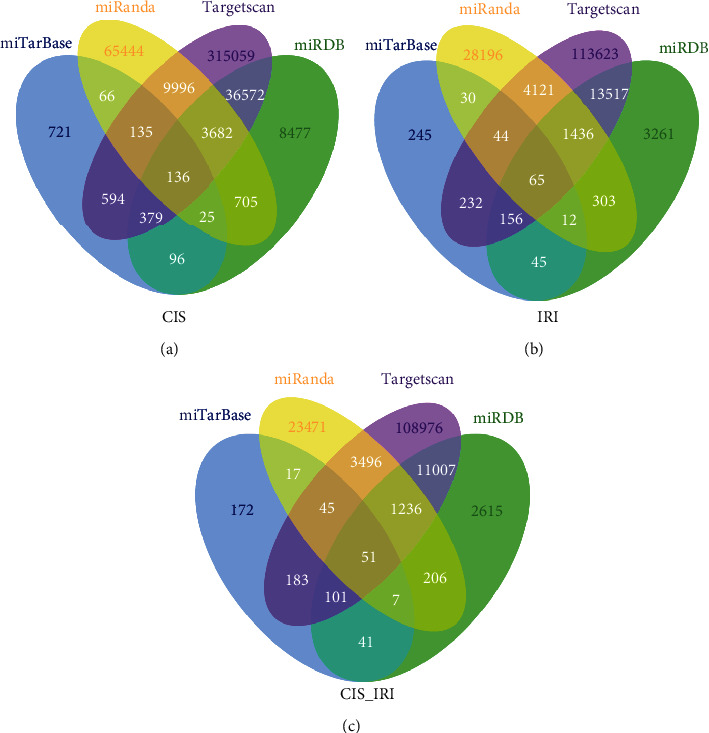
Venn diagram showing the miRNA-mRNA interaction of DEGs in the CIS, IRI and CIS_IRI groups according to four miRNA prediction databases. (a) Predicted miRNA-mRNA interaction of DEGs in the CIS group. (b) Predicted miRNA-mRNA interaction of DEGs in the IRI group. (c) Predicted miRNA-mRNA interaction of DEGs in the CIS_IRI group.

The overlapping DEGs in the CIS and IRI (CIS_IRI) groups and the DEGs unique to the CIS and IRI groups were clarified. The miRNA-mRNA interactions of these three sets of DEGs were obtained by using four databases: miTarBase, miRanda, miRDB, and TargetScan (Figure 2).

**Figure 3:**
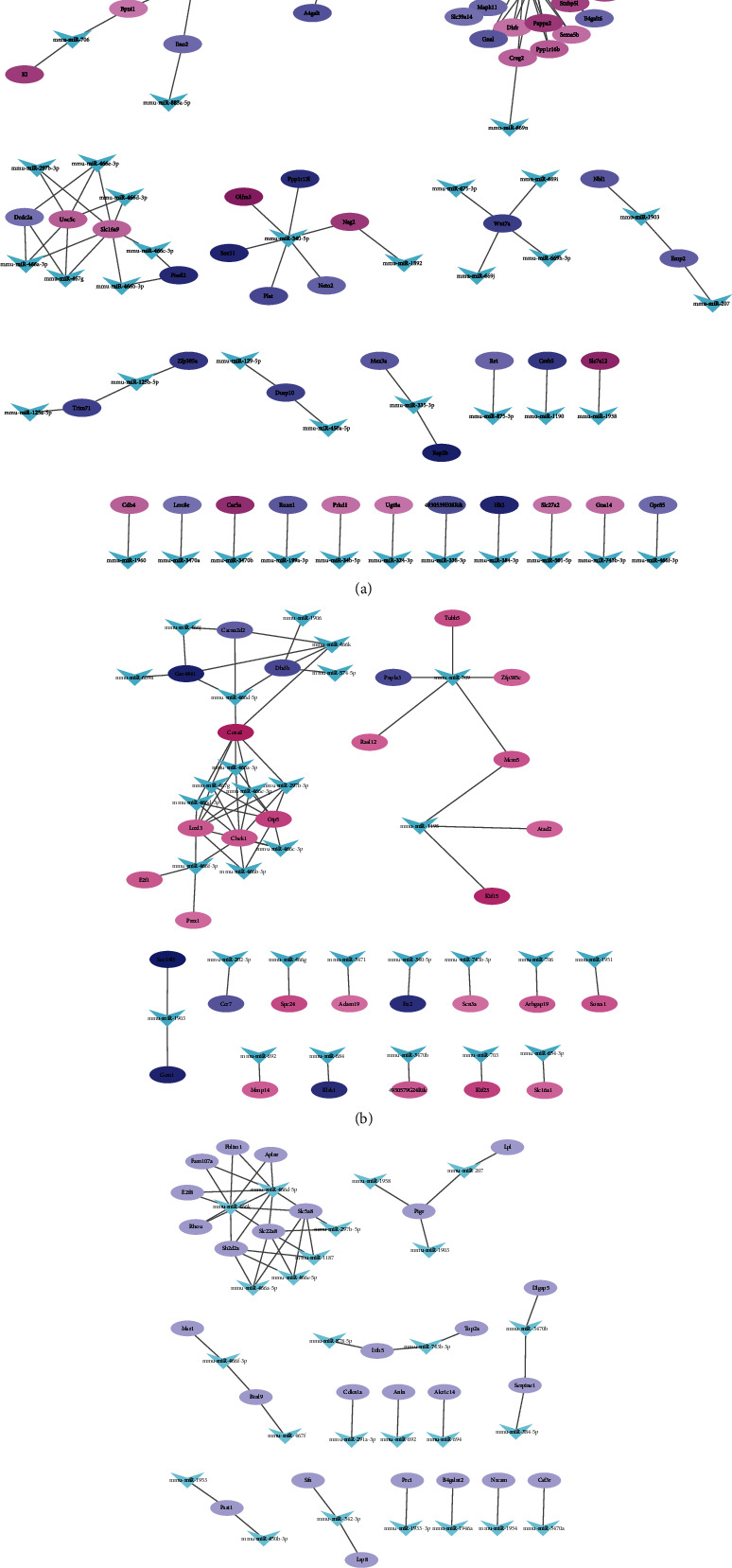
miRNA-mRNA regulatory networks of DEGs overlapping in four miRNA prediction databases. (a) miRNA-mRNA networks of the CIS group. (b) miRNA-mRNA networks of the IRI group. The genes in red represent upregulation, and the genes in green represent downregulation. (c) miRNA (arrow)-mRNA (oval) networks of the CIS_IRI group. Since the expression trend of overlapping DEGs is not consistently in CIS and IRI, DEGs are all in purple.

To obtain a more reliable miRNA-mRNA interaction, we searched the overlapping miRNA-mRNA interactions in the four databases. Three sets of miRNA-mRNA interactions were visualized using Cytoscape. In the CIS group, the four databases had 136 identical miRNA-mRNA interactions, including 64 DEGs. In the network, mmu-miR-466k, mmu-miR-466d-5p, and mmu-miR-340-5p had a higher degree (Figure 3(a)). The IRI group had 65 identical miRNA-mRNA interactions, including 30 DEGs. mmu-miR-709, mmu-miR-466e-3p, mmu-miR-466a-3p, mmu-miR-466f-3p, and mmu-miR-297b-3p target most of the DEGs (Figure 3(b)). The CIS_IRI group had 51 miRNA-mRNA interactions (Figure 3(c)). In the ICS_IRI miRNA-mRNA interactions, the upregulated mRNAs were Pigr, Sh2d2a, Slc5a8, Slc22a8, Akr1c14, Itih5, Btnl9, Fam107a, and Aplnr. The downregulated mRNAs were Sfn, Lrp8, Rhou, B4galnt2, Nrcam, and Csf3r. The expression trends of 11 mRNAs were the complete opposite. They were AnlN, Cdkn1a, Dlgap5, E2f8, Fblim1, Lpl, Msr1, Prc1, Psat1, Serpine1, and Top2a. mmu-miR-466k and mmu-miR-466d-5p are also the top two miRNAs with high degree, followed by mmu-miR-466e-5p, mmu-miR-466a-5p, and mmu-miR-1187.

### 3.3. Functional and Pathway Enrichment Analyses of DEGs

**Figure 4:**
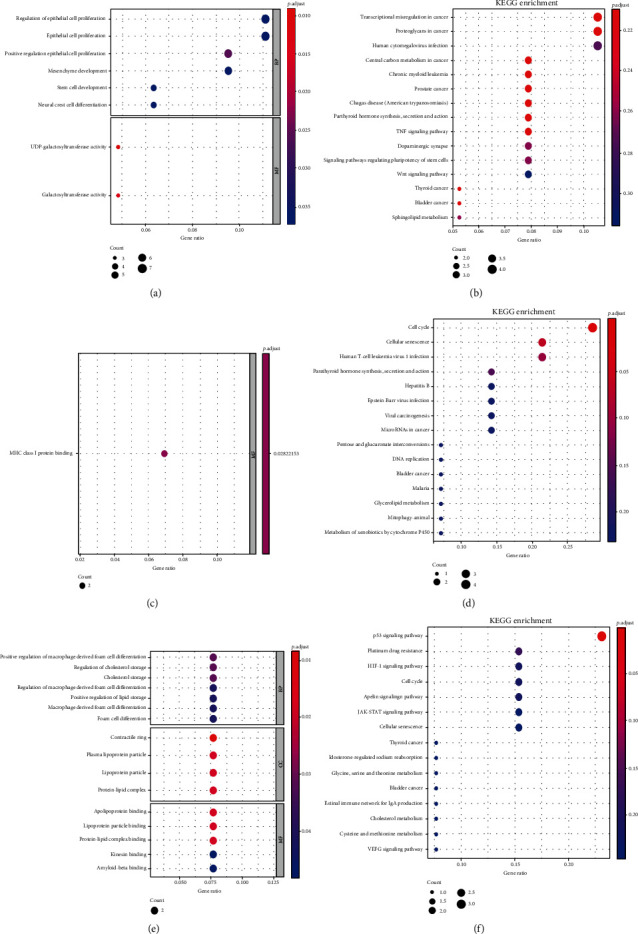
GO and signaling pathway enrichment analysis of DEGs in miRNA-mRNA networks. (a, b) GO enrichment and KEGG analysis of DEGs in the CIS group. (c, d) GO enrichment and KEGG analysis of DEGs in the IRI group. (e, f) GO enrichment and KEGG analysis of DEGs in the CIS_IRI group.

The function and enrichment of the genes in the three miRNA-mRNA regulatory networks of CIS, IRI, and CIS_IRI were analyzed. GO analysis showed that the DEGs in the CIS group were mainly involved in epithelial cell proliferation-related functions (Figure 4(a)). DEGs in the IRI group were mainly involved in MHC class I protein binding (Figure 4(c)). The DEGs in the CIS-IRI group were involved in multiple molecular functions (MFs), biological processes (BPs), and cellular components. Among them, there were significant differences in the contractile ring, plasma lipoprotein particle, lipoprotein particle, protein-lipid complex, apolipoprotein binding, lipoprotein, particle binding, and protein-lipid complex binding (Figure 4(e)).

KEGG analysis showed that the DEGs were mainly enriched in tumor-related pathways and TNF pathways in the CIS group (Figure 4(b)). The DEGs of the IRI group were mainly enriched in the cell cycle and cell senescence pathways (Figure 4(d)). The DEGs of the CIS-IRI group were mainly involved in the p53 signaling pathway, platinum drug resistance, HIF-1 signaling pathway, cell cycle, apelin signaling pathway, JAK-STAT signaling pathway, cellular senescence, and PI3K-Akt signaling pathway (Figure 4(f)). The DEGs of the CIS-IRI group involved in these pathways are Aplnr, Sfn, Csf3r, Cdkn1a, Serpine1, and Top2a.

### 3.4. PPI Network Analysis

**Figure 5:**
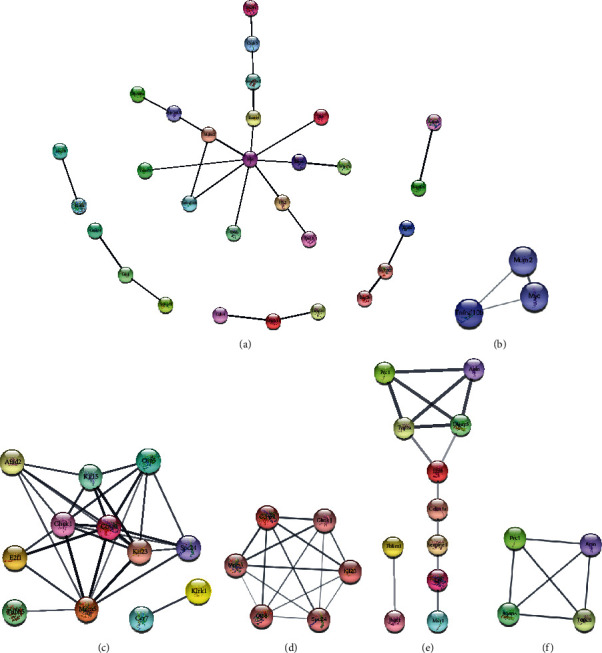
PPI network of the overlapping DEGs in miRNA-mRNA networks. (a) PPI network of the CIS group. (b) Subnetwork of the overlapping DEGs of the CIS group. (c) PPI network of the IRI group. (d) Subnetwork of the overlapping DEGs of the IRI group. Red circles, upregulated genes; blue circles, downregulated genes. (e) PPI network of the CIS_IRI group. (f) Subnetwork of the overlapping DEGs in the CIS_IRI group. The overlapping DEGs showed the opposite trend of expression in the CIS and IRI groups. Since the expression trend of overlapping DEGs is not consistent in CIS and IRI, the color of the DEGs is random.

The PPIs of the DEGs in the miRNA-mRNA networks of the CIS, IRI, and CIS-IRI groups were constructed with the STRING database. The results of CytoHubba analysis showed that the hub gene in the CIS group was Myc and that the genes in the IRI group were Mcm5 and E2f1. Those in the CIS-IRI group were Prc1 and Dlgap5 (Figures 5(a), 5(c), and 5(e)). Subnetwork analysis obtained one significant module network based on MCODE of the PPI network in three groups. All node genes in the modules of the CIS group were downregulated genes (Figure 5(b)), while all node genes in the modules of the IRI group were upregulated genes (Figure 5(d)). Functional enrichment analysis of these genes in the modules showed that the genes in the CIS group were mainly related to the cell cycle, cell senescence, and cell proliferation processes. The genes in the IRI group were mainly related to the cell cycle. Prc1, AnlN, Dlgap5, and Top2a are node genes in the modules of the CIS-IRI group that are mainly related to the cell cycle (Figure 5(e)). Interestingly, these four genes were downregulated in the CIS group and upregulated in the IRI group. Most of the node genes in the modules are target genes of mmu-mir-466 or mmu-mir-709. These results suggest that mmu-mir-466 or mmu-mir-709 may play an important role in the pathological process of AKI.

### 3.5. Expression of DEGs and Key miRNAs in Kidney Tissues from CIS- and IRI-Induced AKI Models

**Figure 6:**
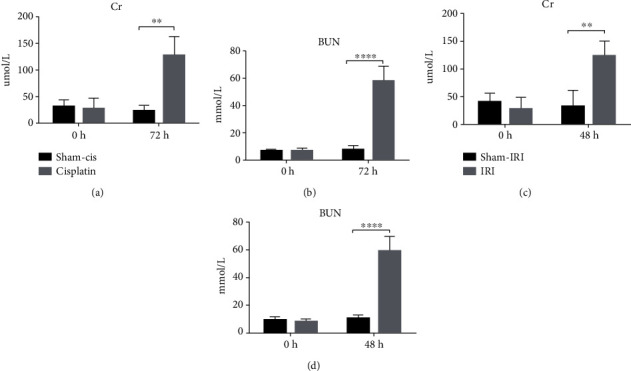
Changes in creatinine and urea nitrogen in CIS-induced AKI and IRI-induced AKI. (a, b) Cr and BUN in CIS-induced AKI models. (c, d) Cr and BUN in IRI-induced AKI models (*n* = 5).

To verify the results of the above analysis, two types of AKI models were established, and renal tissues were extracted for the detection of DEGs and miRNA expression. The observed time points were consistent with the analyzed datasets. The CIS group and the IRI group had time points of 72 h and 48 h, respectively. According to previous studies of animal models, 72 h and 48 h were the times when serum creatinine and blood urea nitrogen (BUN) had the most significant changes in CIS and IRI, respectively. As shown in Figure 6, creatinine and BUN increased significantly in the AKI group.

**Figure 7:**
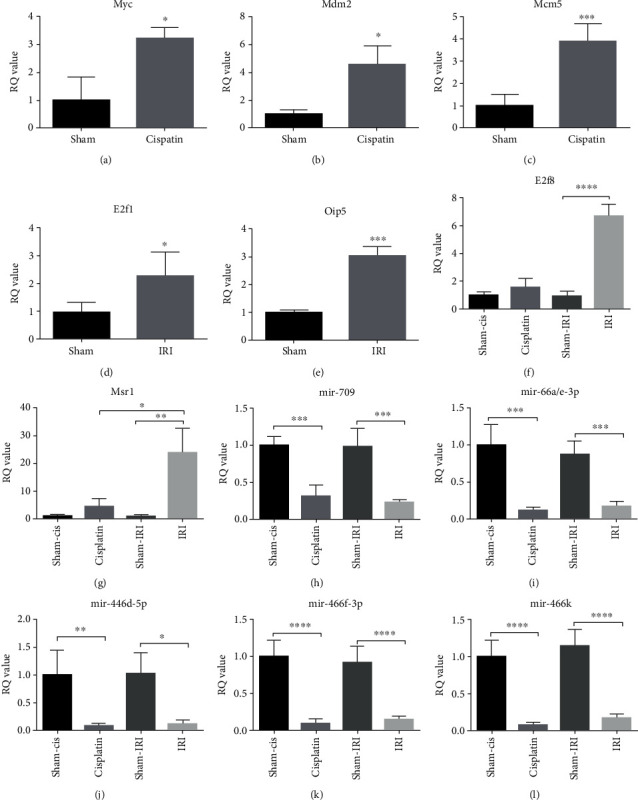
Key genes and miRNA expression in the kidneys of different AKI animal models. (a, b) The expression of DEGs in CIS-induced AKI models. (c–g) The expression of DEGs in IRI-induced AKI models. (h–l) The expression of key miRNAs in the AKI model. ^∗^*P* < 0.05, ^∗∗^*P* < 0.01, ^∗∗∗^*P* < 0.001, and ^∗∗∗∗^*P* < 0.0001 (*n* = 5).

We examined the key node genes associated with mmu-mir-466 or mmu-mir-709 in the CIS and IRI groups. The results showed that the mRNA expression levels of Myc and Mdm2 were significantly increased in the CIS group (Figures 7(a) and 7(b)). In the IRI group, the expression levels of E2f1, Mcm5 and Oip5 were significantly increased (Figures 7(c)–7(e)). For E2f8 and Msr1, which expressed opposite trends in the two groups in the data analysis, E2f8 was not different in the CIS group but was significantly increased in the IRI group (Figure 7(f)). Msr1 was significantly elevated in both the CIS and IRI groups but was more elevated in the IRI group (Figure 7(g)). Although these results were not completely consistent with the results of the analysis, they all suggested that the gene expression associated with the cell cycle changed significantly in both CIS- and IRI-induced AKI. Cell cycle-related changes play an important role in the development of AKI, and this process may be more active in IRI. miRNA-mRNA interaction analysis revealed that Myc, which is regulated by mir-709, was downregulated in CIS. Myc, which was a target of mir-709, was downregulated in the data analysis. Therefore, miR-709 should theoretically be upregulated in CIS due to the negative regulatory effect of miRNA. In fact, we found that the level of mir-709 decreased in the CIS group (Figure 7(h)). This may be the reason for the actual detection of increased Myc expression. For mir-466, miRNA-mRNA analysis showed that most of the target genes of mir-466 were upregulated in both the CIS and IRI groups, so the level of mir-466 should be decreased. The actual detection results are consistent with this inference (Figures 7(i)–7(l)).

## 4. Discussion

Previous studies have shown that proximal tubular epithelial cell injury is the most important pathological feature of AKI [8]. Ischemia and nephrotoxic drugs are the most common causes of AKI, so IRI and CIS injections are the most likely models to simulate the occurrence and development of AKI. The overlapping CIS- and IRI-induced DEGs, such as Cdkn1a, Serpine1, and Top2a, in the main pathways, basically represent the pathological characteristics of AKI. For example, the p53 signaling pathway, cell cycle, and cellular senescence indicate the processes of repair, regeneration, senescence, and apoptosis of renal tubular epithelial cells. Cdkn1a is involved in p53/TP53-mediated inhibition of cellular proliferation in response to DNA damage. Upregulation of p21 encoded by Cdkn1a can induce cell cycle arrest [9]. According to the detection of the p21 levels in the plasma, urine, and organs of the AKI model, Johnson and Zager considered that plasma and urinary p21 can be potential biomarkers of AKI and renal aging [10]. In the kidney, nuclear p21 is located in the proximal and distal tubules. Because of the important role of p21 in the cell cycle, p21 is also considered to be an important marker of renal aging [11]. Because of the involvement of cell cycle arrest, the pathogenesis of AKI can be accompanied by renal senescence, which may be one of the reasons leading to the increase in AKI in the elderly [12]. GO analysis also showed changes in lipid metabolism in AKI due to both causes. This is consistent with the results of previous studies [13]. For example, Lpl and Msr1 are important genes involved in lipid metabolism. Lpl is the main rate-limiting enzyme in the process of high triglyceride metabolism, which plays a role in the metabolism of chylomicron (CM) and very low-density lipoprotein (VLDL) [14]. Studies have also shown that there is a significant decrease in the expression of LPL in CIS-induced AKI [15]. The disorder of lipid metabolism may lead to lipotoxicity to the kidney caused by lipid deposition in renal cells, which leads to injury. Lipid metabolism-related therapies targeting PPAR*α* and other targets can also be one of the treatments for AKI.

The DEGs of AKI caused by the two abovementioned causes are not completely consistent. These DEGs are involved in different pathways, which may indicate that the occurrence and development of AKI due to different causes have their own characteristics. Cisplatin was the first metal complex with anticancer activity and was considered the most promising anticancer therapeutic [16]. Therefore, KEGG analysis showed that the DEGs in the CIS group were mostly related to tumors. Cisplatin is eliminated predominantly by the kidney, and CIS enters renal epithelial cells from the glomerular filtrate. After entering the cell, CIS undergoes metabolic activation to highly reactive molecules, which affects the cellular antioxidant system [17]. As the GO analysis shows, tubular epithelial cells can be the primary site of injury. Although multiple mechanisms contribute to the pathogenesis of CIS nephrotoxicity, evidence suggests that DNA damage plays a critical role [18]. Cisplatin is known to cause inter- and intrastrand DNA crosslinks, which perturb DNA replication and transcription, thereby inducing replication stress, which may eventually result in cell cycle arrest and apoptosis [19]. The hub gene Myc in the CIS group is a transcription factor that mainly activates the transcription factor of growth-related genes and participates in cellular self-renewal [20]. The upregulation of Myc in the actual detection may be due to the fluctuating state of the expression of Myc during the repair of AKI, which will be upregulated after the beginning of the repair process. The c-Myc gene is an important member of the Myc gene family, and studies have shown that c-Myc may have a role in the regulation of tubular cell death during AKI and that overexpression of c-Myc can lead to cell proliferation [21, 22]. Recent studies have shown that Myc activation implicates the pathway in renal fibrosis and in the progression of AKI to CKD [23, 24], and Myc could be a potential therapeutic target in tubulointerstitial diseases [25]. The analysis showed that Myc was downregulated in the CIS group, while the mRNA expression levels of Myc and Mdm2 in the CIS group were significantly increased in the actual test. Generally, it is understandable that the results of the bioinformatics analysis are inconsistent with the results of the validation experiments. For example, the animal models used in the different studies are not completely consistent. Moreover, cisplatin may be obtained from different manufacturers, and there are differences in drug purity. All of these factors may lead to inconsistent degrees of damage in the models, which could result in the occurrence of different repair stages at the same time. Both Myc and Mdm2 can promote cell proliferation, which indicates that our model may be in the proliferation and repair stage. The model in the CIS dataset may be in the damage stage, in which most differentially expressed genes have low expression. Due to the cytotoxicity of CIS, various biological functions of cells may be inhibited. This may be why most of the DEGs in the CIS group, including the genes above, were downregulated by CIS injection in the dataset analysis. In terms of the level of expression, future experiments can be performed to analyze the samples at different time points to determine the change trend of gene expression.

GO analysis showed that DEGs in the IRI group could participate in the MHC class I protein binding process. The function of MHC-I was to present intrinsic antigens and activate CD8^+^ T cells. This indicates that the change in immune function is one of the important characteristics in renal IRI. Interstitial inflammatory responses, including increasingly complex T- and B-cell populations, highlight the continuing kidney pathology after AKI, and numerous studies have examined the effects of T and B lymphocytes on IRI-induced AKI [26, 27]. The hub gene Mcm5 is a component of the Mcm2-7 complex, which is the putative replicative helicase essential for DNA replication initiation and elongation in eukaryotic cells [28]. E2f1 is a transcription activator involved in cell cycle regulation or DNA replication [29]. In the subnetwork, Oip5, which is required for the progression of mitosis [30], also has a high degree according to the CytoHubba analysis. These DEGs were all related to the cell cycle or proliferation. PPI analysis showed that most of the DEGs in the IRI group were upregulated, which was different from those in the CIS group. At the peak of IRI damage, most of the upregulated DEGs were related to the cell cycle. This reflects that the repair process of IRI may already be active during the period of maximum change in biochemical index and that the repair ability is strong.

miRNAs are noncoding RNA molecules of 21–25 nucleotides that regulate gene expression through the posttranscriptional repression of their target mRNAs [31]. They can inhibit target mRNA translation or induce target mRNA degradation through partial or full complementarity to the 3′ untranslated region of their target mRNAs. Evidence suggests that most genes are regulated by miRNAs. A miRNA may regulate different genes and vice versa. In the kidney, miRNA plays a key role in both physiological and pathological activities. Several studies have shown that at least a dozen kinds of miRNAs may play an important role in the pathogenesis of AKI induced by IRI and CIS [32]. miRNA may become a new marker or new treatment. In our study, miRNA-mRNA interaction analysis showed that miR-466 and miR-709 have a high degree. At present, some studies have clarified the expression changes or effects of these two miRNAs in pathological states. In the study of tumors, the role of mir-466 is considered to inhibit the proliferation and invasion of carcinoma [33]. In the kidney, miR-466 may also be related to osmoregulation and urine concentration in mice [34]. In the case of injury, miR-466 was significantly downregulated in the livers of the IRI model animals [35]. This is consistent with the results detected in the CIS and IRI models. During injury, the kidney promoted damage repair by reducing mir-466 production and increasing the expression of its downstream genes associated with cell proliferation. mir-709 has been identified as a nuclear-enriched microRNA that can control the biogenesis of other miRNAs by directly targeting their primary transcripts in the nucleus [36]. Some studies have shown that miR-709 inhibits adipocyte differentiation or modulates the inflammatory response by targeting the GSK3*β*/Wnt signaling pathway [37, 38]. In a recent study, Guo et al. confirmed that in a CIS-induced AKI mouse model and in biopsy samples of human AKI kidney tissue, miR-709 was significantly upregulated in proximal tubular cells (PTCs) [39]. Their results suggest that miR-709 has an important role in mediating AKI via negative regulation of mitochondrial transcription factor (TFAM) and subsequent mitochondrial dysfunction. This is also consistent with the analysis results of this study. Our detection results of the expression of miR-709 are inconsistent with their study and may be related to the degree of damage in the AKI model. However, at present, tumor research is primarily focused on the roles of mir-466 and miR-709, and the specific roles in AKI need to be further studied.

## 5. Conclusion

After analyzing the GEO database, DEGs were extracted from different AKI models for GO and KEGG analyses. It was concluded that apoptosis was dominant in CIS-induced AKI lesions and that IRI is characterized by an increasing inflammatory response of the system accompanied by local kidney injury. Combining the construction of PPI networks and miRNA-mRNA networks, we identified possible key genes and miRNAs as Myc, Mcm5, E2f1, Oip5, Mdm2, E2f8, mir-466, and mir-709. These node genes and miRNAs are all associated with the cell repair regeneration process. We established the AKI model, and the expression of these key genes and miRNAs in the CIS- or IRI-induced AKI model was basically consistent with the data analysis. Since mir-466 and mir-709 have not been studied deeply in kidney disease, their role in AKI needs further investigation. These DEGs and miRNAs can serve as targets for future AKI therapy.

## Data Availability

The data used to support the findings of this study are included within the article.

## References

[1] Myers P. S., McNeely M. E., Pickett K. A., Duncan R. P., Earhart G. M. (2017). Acute kidney disease and renal recovery: consensus report of the Acute Disease Quality Initiative (ADQI) 16 workgroup. *Nature reviews Nephrology*.

[2] Zuk A., Bonventre J. V. (2016). Acute kidney injury. *Annual review of medicine*.

[3] Bartel D. P. (2009). MicroRNAs: target recognition and regulatory functions. *Cell*.

[4] Liu J., Kumar S., Dolzhenko E., Alvarado G. F., McMahon A. P. (2017). Molecular characterization of the transition from acute to chronic kidney injury following ischemia/reperfusion. *Jci Insight*.

[5] Späth M. R., Bartram M. P., Palacio-Escat N. (2019). The proteome microenvironment determines the protective effect of preconditioning in cisplatin-induced acute kidney injury. *Kidney international*.

[6] de Almeida D. C., Bassi Ê. J., Azevedo H. (2016). A regulatory miRNA-mRNA network is associated with tissue repair induced by mesenchymal stromal cells in acute kidney injury. *Frontiers in immunology*.

[7] Lee C. G., Kim J. G., Kim H. J. (2014). Discovery of an integrative network of microRNAs and transcriptomics changes for acute kidney injury. *Kidney international*.

[8] Thadhani R., Pascual M., Bonventre J. V. (1996). Acute renal failure. *The New England journal of medicine*.

[9] Deng C., Zhang P., Harper J. W., Elledge S. J., Leder P. (1995). Mice lacking p21 ^_CIP1/WAF1_^ undergo normal development, but are defective in G1 checkpoint control. *Cell*.

[10] Johnson A. C., Zager R. A. (2018). Plasma and urinary p21: potential biomarkers of AKI and renal aging. *American Journal of Physiology. Renal Physiology*.

[11] Docherty M. H., O'Sullivan E. D., Bonventre J. V., Ferenbach D. A. (2019). Cellular senescence in the kidney. *Journal of the American Society of Nephrology*.

[12] Chao C. T., Wang J., Wu H. Y., Huang J. W., Chien K. L. (2018). Age modifies the risk factor profiles for acute kidney injury among recently diagnosed type 2 diabetic patients: a population-based study. *Geroscience*.

[13] Erpicum P., Rowart P., Defraigne J. O., Krzesinski J. M., Jouret F. (2018). What we need to know about lipid-associated injury in case of renal ischemia-reperfusion. *American Journal of Physiology-Renal Physiology*.

[14] Eckel R. H. (1989). Lipoprotein lipase. A multifunctional enzyme relevant to common metabolic diseases. *The New England journal of medicine*.

[15] Li S., Nagothu K., Ranganathan G. (2012). Reduced kidney lipoprotein lipase and renal tubule triglyceride accumulation in cisplatin-mediated acute kidney injury. *American Journal of Physiology. Renal Physiology*.

[16] Hambley T. W. (2007). Developing new metal-based therapeutics: challenges and opportunities. *Dalton Transactions*.

[17] Perše M., Večerić-Haler Ž. (2018). Cisplatin-induced rodent model of kidney injury: characteristics and challenges. *BioMed Research International*.

[18] Dasari S., Tchounwou P. B. (2014). Cisplatin in cancer therapy: molecular mechanisms of action. *European Journal of Pharmacology*.

[19] Zhu S., Pabla N., Tang C., He L., Dong Z. (2015). DNA damage response in cisplatin-induced nephrotoxicity. *Archives of Toxicology*.

[20] Seruggia D., Oti M., Tripathi P. (2019). TAF5L and TAF6L maintain self-renewal of embryonic stem cells via the MYC regulatory network. *Molecular Cell*.

[21] Bao H., Ge Y., Wang Z. (2014). Delayed administration of a single dose of lithium promotes recovery from AKI. *Journal of the American Society of Nephrology*.

[22] Ortiz A., Lorz C., Catalán M. P. (2000). Expression of apoptosis regulatory proteins in tubular epithelium stressed in culture or following acute renal failure. *Kidney international*.

[23] Venkatachalam M. A., Weinberg J. M., Kriz W., Bidani A. K. (2015). Failed tubule recovery, AKI-CKD transition, and kidney disease progression. *Journal of the American Society of Nephrology*.

[24] Hultström M., Becirovic-Agic M., Jönsson S. (2018). Comparison of acute kidney injury of different etiology reveals in-common mechanisms of tissue damage. *Physiological Genomics*.

[25] Lemos D. R., McMurdo M., Karaca G. (2018). Interleukin-1*β*Activates a MYC-dependent metabolic switch in kidney stromal cells necessary for progressive tubulointerstitial fibrosis. *Journal of the American Society of Nephrology*.

[26] Jang H. R., Gandolfo M. T., Ko G. J., Satpute S. R., Racusen L., Rabb H. (2010). B cells limit repair after ischemic acute kidney injury. *Journal of the American Society of Nephrology*.

[27] Kinsey G. R., Sharma R., Huang L. (2009). Regulatory T cells suppress innate immunity in kidney ischemia-reperfusion injury. *Journal of the American Society of Nephrology*.

[28] Labib K., Tercero J. A., Diffley J. F. (2000). Uninterrupted MCM2-7 function required for DNA replication fork progression. *Science*.

[29] Porse B. T., Pedersen T. A., Xu X. (2001). E2F repression by C/EBP*α* is required for adipogenesis and granulopoiesis in vivo. *Cell*.

[30] Nardi I. K., Zasadzińska E., Stellfox M. E., Knippler C. M., Foltz D. R. (2016). Licensing of centromeric chromatin assembly through the Mis18*α*-Mis18*β* heterotetramer. *Molecular Cell*.

[31] Trionfini P., Benigni A., Remuzzi G. (2015). MicroRNAs in kidney physiology and disease. *Nature reviews Nephrology*.

[32] Liu Z., Wang S., Mi Q. S., Dong Z. (2016). MicroRNAs in pathogenesis of acute kidney injury. *Nephron*.

[33] Colden M., Dar A. A., Saini S. (2018). MicroRNA-466 inhibits tumor growth and bone metastasis in prostate cancer by direct regulation of osteogenic transcription factor RUNX2. *Cell Death & Disease*.

[34] Luo Y., Liu Y., Liu M. (2014). Sfmbt2 10th intron-hosted miR-466(a/e)-3p are important epigenetic regulators of Nfat5 signaling, osmoregulation and urine concentration in mice. *Biochimica et Biophysica Acta*.

[35] Zhang L., Song Y., Chen L. (2019). MiR-20a-containing exosomes from umbilical cord mesenchymal stem cells alleviates liver ischemia/reperfusion injury. *Journal of Cellular Physiology*.

[36] Tang R., Li L., Zhu D. (2012). Mouse miRNA-709 directly regulates miRNA-15a/16-1 biogenesis at the posttranscriptional level in the nucleus: evidence for a microRNA hierarchy system. *Cell research*.

[37] Chen H., Mo D., Li M. (2014). miR-709 inhibits 3T3-L1 cell differentiation by targeting GSK3*β* of Wnt/*β*-catenin signaling. *Cellular Signalling*.

[38] Li M., Chen H., Chen L., Chen Y., Liu X., Mo D. (2016). miR-709 modulates LPS-induced inflammatory response through targeting GSK-3beta. *International immunopharmacology*.

[39] Guo Y., Ni J., Chen S. (2018). MicroRNA-709 mediates acute tubular injury through effects on mitochondrial function. *Journal of the American Society of Nephrology*.

